# How peer behavior shapes academic misconduct: The roles of moral disengagement and shame

**DOI:** 10.1371/journal.pone.0358871

**Published:** 2026-10-14

**Authors:** Yang Yang, Lanxin Hong

**Affiliations:** Dongshin University, Naju, Republic of Korea; Institute of Medical Biochemistry Leopoldo de Meis (IBqM) - Federal University of Rio de Janeiro (UFRJ), BRAZIL

## Abstract

Academic misconduct predisposition remains an important concern in higher education, and peer behavior perception has emerged as a relevant correlate of students’ academic integrity-related tendencies. Drawing on social cognitive theory and Bandura’s moral disengagement theory, this study examined how perceived peer misconduct is associated with students’self-reported predisposition toward academic misconduct through moral disengagement and shame. Data were collected through a cross-sectional survey of 418 undergraduate students from a comprehensive university in Shandong, China. Structural equation modeling and Bootstrap analyses with 5,000 resamples were used to estimate a serial mediation model. The results showed that peer behavior perception was positively associated with moral disengagement (β = .423, p < .001), moral disengagement was negatively associated with shame (β = −.223, p < .001), and shame was negatively associated with academic misconduct predisposition (β = −.309, p < .001). The direct association between peer behavior perception and academic misconduct predisposition remained statistically significant (β = .272, p < .001). Bootstrap results further showed significant indirect effects through moral disengagement, through shame, and through the serial pathway from moral disengagement to shame. The serial indirect effect was significant, effect = .026, 95% CI [.01, .04]. These findings suggest that perceived peer misconduct is associated with academic misconduct predisposition partly through higher moral disengagement and lower shame. The study provides a theory-consistent account of how perceived peer norms, moral rationalization, and affective self-sanctions are related to academic integrity among university students.

## 1 Introduction

Academic integrity remains an important concern in higher education because it is closely related to the quality of learning, institutional trust, and students’ ethical development. Recent evidence from Chinese higher education suggests that academic integrity continues to require scholarly and practical attention. For example, a study involving students from 63 Chinese higher education institutions found that Chinese college students’ integrity was at a medium level, indicating the continued need to strengthen integrity cultivation in universities [1]. Similarly, research on Chinese university students has shown that academic dishonesty remains an important issue at the university level and is associated with students’ individual characteristics and rule-related attitudes [2]. In the Chinese context, the expansion of higher education and intensified academic competition have further made academic dishonesty a salient issue for educators and researchers [3]. Although existing studies have examined a wide range of antecedents of academic dishonesty, further attention is still needed to clarify the psychological processes through which perceived peer misconduct is associated with students’ own predisposition to engage in academic misconduct.

The scholarly literature has generally examined academic dishonesty from individual, situational, and integrative perspectives. Individual-level explanations emphasize factors such as moral reasoning, academic pressure, achievement motivation, and personality-related characteristics [4,5]. Situational explanations focus on contextual influences, including institutional climate, opportunity structures, and peer influence [6,7]. Integrative perspectives have attempted to connect these individual and contextual factors by examining how students interpret and respond to academic environments [8–10]. Within this body of research, peer behavior perception has emerged as a particularly important correlate of students’ academic dishonesty tendencies.

Peer behavior perception refers to students’ perceptions of how frequently their peers engage in academically dishonest conduct. Social cognitive theory suggests that individuals learn not only from direct experience but also from observing social models and interpreting normative cues in their environment [11]. In academic settings, perceived peer misconduct may shape students’ understanding of what is common, tolerated, or expected. When students believe that many classmates cheat, plagiarize, or violate academic rules, such perceptions may be associated with a lower personal threshold for judging similar conduct as unacceptable [12–14]. However, the psychological mechanisms linking perceived peer misconduct to students’ own predisposition toward academic misconduct require further clarification.

Moral disengagement provides a useful theoretical lens for understanding this process. Bandura’s theory of moral disengagement describes how individuals selectively deactivate moral self-regulatory processes through mechanisms such as moral justification, euphemistic labeling, advantageous comparison, displacement of responsibility, diffusion of responsibility, and minimization of harm [15,16]. In academic contexts, students may frame plagiarism as “collaborative referencing,” view cheating as a response to unfair academic pressure, or justify dishonest behavior by assuming that “everyone does it” [17]. Perceived peer misconduct may provide a normative context in which such rationalizations become more psychologically available. Prior studies in organizational and educational settings have shown that moral disengagement is associated with unethical conduct and academic cheating tendencies [18–21].

A more accurate account of moral disengagement theory also requires attention to affective self-sanctions. Bandura’s theory does not treat moral disengagement as a purely cognitive process. Rather, it explains how cognitive restructuring weakens the self-evaluative and emotional restraints that normally inhibit harmful or unethical conduct [16,22]. Shame is one such moral emotion. It is commonly understood as a negative self-evaluative emotion that can arise when individuals judge themselves as having violated moral or social standards [23–25]. In relation to academic misconduct, anticipated shame may be associated with a lower willingness to engage in dishonest academic practices because it represents an internal emotional cost of wrongdoing.

The relationship between peer behavior perception, moral disengagement, and shame can therefore be understood as a sequential psychological process. When students perceive academic misconduct as common among peers, they may be more likely to adopt justifications that reduce moral self-sanction. These justifications may then be associated with lower shame regarding academic dishonesty, which in turn may relate to a stronger predisposition toward academic misconduct. This interpretation is consistent with moral emotion theory, which emphasizes the role of moral emotions in ethical judgment and conduct [26], and with studies suggesting that social norms and moral emotions are closely connected in the domain of academic integrity [27–30].

It is also important to clarify the nature of the outcome examined in the present study. The current research does not assess verified behavioral records of academic misconduct or directly observed misconduct events. Instead, it examines students’ self-reported predisposition, tendency, or likelihood of engaging in academic misconduct. This distinction is important because attitudes, intentions, and self-reported tendencies are related to but not equivalent to actual behavioral outcomes. Accordingly, the present study focuses on psychological associations with academic misconduct predisposition rather than making claims about confirmed misconduct behavior.

Based on the above theoretical framework, this study examines a two-stage mediation model among 418 Chinese college students. We investigate whether peer behavior perception is associated with stronger moral disengagement (H1), whether moral disengagement is associated with lower shame (H2), and whether reduced shame is associated with a higher predisposition toward academic misconduct (H3). Structural equation modeling and Bootstrap methods are used to test the proposed serial mediation pathway. By situating shame within Bandura’s moral disengagement framework, this study aims to provide a more theoretically accurate account of how perceived peer misconduct is linked to students’ academic misconduct predisposition through moral self-regulatory and affective self-sanction processes.

## 2 Methods

### 2.1 Research design

This study employed a cross-sectional survey design. Given the sensitive nature of academic misconduct, procedural remedies were implemented to minimize potential social desirability bias and common method variance. A pilot study (n = 50) was conducted prior to the formal administration to refine the questionnaire items based on participant feedback, ensuring clarity and cultural appropriateness. This study was conducted in accordance with the Declaration of Helsinki and was approved by the Ethics Committee of Shandong Normal University (Approval Code: SDNUTYDW2025057; Approval Date: 27 Aug 2025). Written informed consent was obtained from all participants prior to participation in the study.

### 2.2 Participants

A stratified sampling procedure was used to recruit undergraduate students from humanities, sciences, and engineering programs at a comprehensive university in Shandong, China. The survey was administered between August 30, 2025, and January 10, 2026. To reach students from different academic disciplines and grade levels, the questionnaire link was distributed by student counselors through class communication groups. Prior to data collection, all participants were informed about the research purpose, the voluntary nature of their participation, and the strict confidentiality of their responses. Participants were required to sign an electronic informed consent form and were assured that they could withdraw from the survey at any time without penalty.

A total of 450 questionnaires were distributed, and 418 valid responses were obtained, yielding a valid response rate of 92.9%. The final sample consisted of 216 males (51.7%) and 202 females (48.3%). In terms of grade level, the sample included 109 freshmen (26.1%), 106 sophomores (25.4%), 101 juniors (24.2%), and 102 seniors (24.4%). Regarding academic discipline, 126 students were enrolled in humanities programs (30.1%), 148 in science programs (35.4%), and 144 in engineering programs (34.4%). The sample therefore included students from the main disciplinary categories and all four undergraduate grade levels covered by the study design.

### 2.3 Measurement tools

Because the original measurement tools were developed in English, a standard translation and back-translation procedure [31] was adopted to create the Chinese versions of the scales. Two bilingual psychology researchers independently translated the items into Chinese, and another two bilingual experts translated them back into English to ensure conceptual equivalence.

#### 2.3.1 Peer behavior perception scale.

Students’ perceptions of the prevalence of academic misconduct among their peers were assessed using the 8-item scale developed [32]. Sample items include “My classmates often cheat during exams” and “My classmates often plagiarize other people's assignments.” Responses were recorded on a 7-point Likert scale ranging from 1 (strongly disagree) to 7 (strongly agree). This scale has demonstrated robust psychometric properties in international studies [12] and has been validated among Chinese college students [33]. In the current study, the Cronbach's α for this scale was 0.927.

#### 2.3.2 Academic misconduct predisposition scale.

The tendency to engage in academic misconduct was measured using a 12-item scale developed [34]. This instrument evaluates the frequency of various dishonest behaviors over the past year, covering dimensions such as exam cheating, assignment plagiarism, and improper citation. Sample items include “I cheat during exams by looking at other people's answers” and “I collaborate with others on personal assignments without permission.” Participants rated the items on a 5-point Likert scale ranging from 1 (never) to 5 (often). This scale has been widely utilized in academic integrity literature [35]. The Cronbach's α in the present sample was 0.941.

#### 2.3.3 Moral disengagement scale.

The extent to which individuals cognitively rationalize unethical behavior was evaluated using the 8-item Moral Disengagement Scale developed by Moore et al. (2012). Sample items include “As long as no one is harmed, it's okay to do things that are typically considered wrong” and “Compared to others, violating some rules is not a big issue.” Items were rated on a 7-point Likert scale ranging from 1 (strongly disagree) to 7 (strongly agree). This scale is widely applied in organizational and moral psychology research [19]. The Cronbach's α for this scale in the current study was 0.904.

#### 2.3.4 Shame scale.

Students’ emotional responses to academic dishonesty were measured using a 6-item scale adapted [24]. The items assess feelings of shame specifically contextualized within academic behaviors. Sample items include “If I cheated in my studies, I would feel ashamed of myself” and “Engaging in academic misconduct would make me feel like a bad person.” Participants responded on a 7-point Likert scale from 1 (strongly disagree) to 7 (strongly agree). This instrument has shown excellent psychometric properties in previous moral emotion literature [36]. In this study, the Cronbach's α was 0.898.

#### 2.3.5 Control variables.

Consistent with previous empirical studies on academic integrity, gender, grade, major type, and academic performance were included as covariates in the analyses. Academic performance was assessed via self-report, where participants indicated their average grade score from the previous semester on a standard 100-point scale.

### 2.4 Data analysis strategy

Data screening and primary analyses were conducted using R. Descriptive statistics and Pearson correlation analyses were first performed to describe the main variables and examine their bivariate associations. Statistical significance was evaluated using two-tailed tests with α = .05.

Common method bias was assessed using Harman’s single-factor test and a common latent factor approach. For Harman’s single-factor test, all measurement items were entered into an exploratory factor analysis, and the proportion of variance explained by the first unrotated factor was examined. For the common latent factor approach, a latent method factor was added to the measurement model, and the change in model fit and the variance attributable to the common factor were inspected.

Confirmatory factor analysis was conducted to examine the measurement model. The hypothesized four-factor model, including peer behavior perception, moral disengagement, shame, and academic misconduct predisposition, was compared with alternative one-factor, two-factor, and three-factor models. Model fit was evaluated using χ²/df, CFI, TLI, RMSEA, and SRMR. Reliability and convergent validity were examined using Cronbach’s α, composite reliability, average variance extracted, and standardized factor loadings.

Structural equation modeling was used to test the revised serial mediation model. The model estimated the associations from peer behavior perception to moral disengagement, from moral disengagement to shame, and from shame to academic misconduct predisposition. Direct paths from peer behavior perception to shame and academic misconduct predisposition, as well as from moral disengagement to academic misconduct predisposition, were also estimated. Gender, grade, major type, and academic performance were included as control variables. The Bootstrap method with 5,000 resamples was used to estimate indirect effects and 95% confidence intervals.

Additional robustness checks were conducted. Propensity score matching was used to compare students with higher and lower peer behavior perception after balancing observed background variables. Omitted-variable sensitivity analysis was conducted following Cinelli and Hazlett’s framework. Subsample analyses were conducted by gender, grade level, and academic discipline. The exploratory interaction analysis between moral disengagement and shame was reported in S1 Appendix.

## 3 Results

### 3.1 Common method bias test

The common method bias results are presented in Table 1. Harman’s single-factor test showed that the first factor accounted for 36.10% of the total variance. The common latent factor analysis showed ΔCFI = − .044, and the variance attributable to the common factor was 9.00%.

**Table 1:** Common method bias test results.

Inspection Method	Test results	Criteria for judgment	Conclusion
Harman's Single Factor Test	The first factor explains 36.1% of the variation	<50%	Pass
Common Latent Factor Test	ΔCFI = − .044	<0.05	Pass
CLF Test (AVE)	Variation Explained = 9%	<25%	Pass

### 3.2 Descriptive statistics and correlation analysis

Table 2 reports the descriptive statistics and correlations among the study variables. Peer behavior perception was positively correlated with moral disengagement, r = .414, p < .001, and academic misconduct predisposition, r = .499, p < .001. Peer behavior perception was negatively correlated with shame, r = −.314, p < .001. Moral disengagement was positively correlated with academic misconduct predisposition, r = .505, p < .001, and negatively correlated with shame, r = −.341, p < .001. Shame was negatively correlated with academic misconduct predisposition, r = −.507, p < .001.

**Table 2:** Descriptive statistics and correlation analysis results.

Variable	M	SD	1	2	3	4	5	6	7	8
1. Gender	1.48	.50	1							
2. Grade	2.47	1.12	− .037	1						
3. Major type	2.04	.804	− .022	− .020	1					
4. Academic performance	75.05	11.89	.010	.004	.457***	1				
5. Peer Behavior Perception	3.79	.96	− .031	− .039	−.039	− .250***	1			
6. Moral Disengagement	3.71	.86	.064	.189***	−.112*	− .139**	.414***	1		
7. Shame	3.60	.91	.022	− .151**	.082	.129**	− .314***	− .341***	1	
8. Academic misconduct predisposition	3.16	.84	− .007	.143**	− .132**	− .247***	.499***	.505***	− .507***	1

Note: *p < 0.05, **p < 0.01, ***p < 0.001.

### 3.3 Measurement model fit

The CFA results are shown in Table 3. The hypothesized four-factor model showed good fit to the data, χ² = 661.70, df = 521, χ²/df = 1.27, CFI = .984, TLI = .983, RMSEA = .025, and SRMR = .039. The alternative one-factor, two-factor, and three-factor models showed poorer fit than the hypothesized four-factor model.

**Table 3:** Comparison of confirmatory factor analysis models.

Model	χ²	df	χ²/df	CFI	TLI	RMSEA	SRMR
M1:Single factor model	3992.602	527	7.576	.606	.581	.125	.123
M2: Two factor model	2787.441	526	5.299	.743	.726	.101	.111
M3: Three factor model	1903.212	524	3.632	.843	.832	.079	.094
M4: Four factor model	661.701	521	1.270	.984	.983	.025	.039

Note: M1 combines all variables into one factor; M2 combines peer behavior perception with moral disengagement, and shame with academic misconduct; M3 combines peer behavior perception and moral disengagement into one factor; M4 is the hypothesized four-factor model.

Table 4 reports the reliability and convergent validity indices for the four measurement scales. The standardized factor loadings ranged from .623 to .844, indicating that all retained items loaded adequately on their corresponding constructs. Cronbach’s α values ranged from .898 to .941, and composite reliability values ranged from .899 to .942. The AVE values ranged from .545 to .617. These indices were consistent with acceptable internal consistency and convergent validity for peer behavior perception, moral disengagement, shame, and academic misconduct predisposition.

**Table 4:** Measurement model reliability and validity test results.

Item	Question items	Factor loading	t	Cronbach's α	CR	AVE
Peer Behavior Perception	PBP1	.844	51.639	.927	.928	.617
	PBP2	.807	42.058			
	PBP3	.794	39.485			
	PBP4	.692	25.231			
	PBP5	.786	37.966			
	PBP6	.792	39.015			
	PBP7	.806	41.9558			
	PBP8	.746	31.594			
Moral Disengagement	MD1	.697	25.106	.904	.905	.545
	MD2	.694	24.834			
	MD3	.740	29.748			
	MD4	.728	28.395			
	MD5	.779	35.299			
	MD6	.775	34.704			
	MD7	.679	23.411			
	MD8	.793	37.587			
Shame	SH1	.737	29.079	.898	.899	.597
	SH2	.753	31.026			
	SH3	.778	34.608			
	SH4	.777	34.422			
	SH5	.791	36.648			
	SH6	.795	37.342			
Academic Misconduct Predisposition	AMP1	.699	26.451	.941	.942	.579
	AMP2	.792	40.251			
	AMP3	.623	19.896			
	AMP4	.782	38.248			
	AMP5	.803	42.559			
	AMP6	.814	45.228			
	AMP7	.776	37.103			
	AMP8	.754	33.454			
	AMP9	.770	36.016			
	AMP10	.837	51.638			
	AMP11	.782	38.261			
	AMP12	.640	21.127			

Note: All factor loadings are significant and exceed the minimum standard of 0.6; CR is Composite Reliability; AVE is Average Variance Extracted.

### 3.4 Serial mediation model

Table 5 presents the structural paths and Bootstrap indirect effects of the serial mediation model. Peer behavior perception was positively associated with moral disengagement, β = .423, SE = .040, p < .001, 95% CI [.34, .50]. Moral disengagement was negatively associated with shame, β = −.223, SE = .052, p < .001, 95% CI [−.32, −.12]. Peer behavior perception was also negatively associated with shame, β = −.217, SE = .055, p < .001, 95% CI [−.33, −.11].

**Table 5:** Structural paths and bootstrap indirect effects of the serial mediation model.

Path A	Standardized coefficient	SE	t	p	95% CI
PBP → MD	.423	.040	10.583	< .001	[.34, .50]
MD → SH	− .223	.052	−4.306	< .001	[- .32, - .12]
PBP → SH	− .217	.055	−3.959	< .001	[- .33, - .11]
MD → AMP	.262	.042	6.265	< .001	[.18, .34]
SH → AMP	− .309	.038	−8.124	< .001	[- .38, - .24]
PBP → AMP	.272	.044	6.154	< .001	[.18, .36]
Path B					
PBP → MD → AMP	.097	.019	5.028	< .001	[.06, .14]
PBP → SH → AMP	.059	.017	3.439	< .001	[.03, .09]
PBP → MD → SH → AM	.026	.008	3.364	< .001	[.01, .04]
Total indirect effects	.181	.025	7.185	< .001	[.13, .23]
Direct effect	.238	.039	6.122	< .001	[.16, .31]
Total effect	.418	.037	11.274	< .001	[.35, .49]

Note: PBP = peer behavior perception; MD = moral disengagement; SH = shame; AMP = academic misconduct predisposition. Correlations are reported in the lower triangle. Effect ratio = Specific effect/Total effect × 100%.

For academic misconduct predisposition, moral disengagement showed a positive association, β = .262, SE = .042, p < .001, 95% CI [.18, .34]. Shame showed a negative association, β = −.309, SE = .038, p < .001, 95% CI [−.38, −.24]. The direct association between peer behavior perception and academic misconduct predisposition was also statistically significant, β = .272, SE = .044, p < .001, 95% CI [.18, .36]. The serial mediation model is displayed in Fig 1

**Fig 1:**
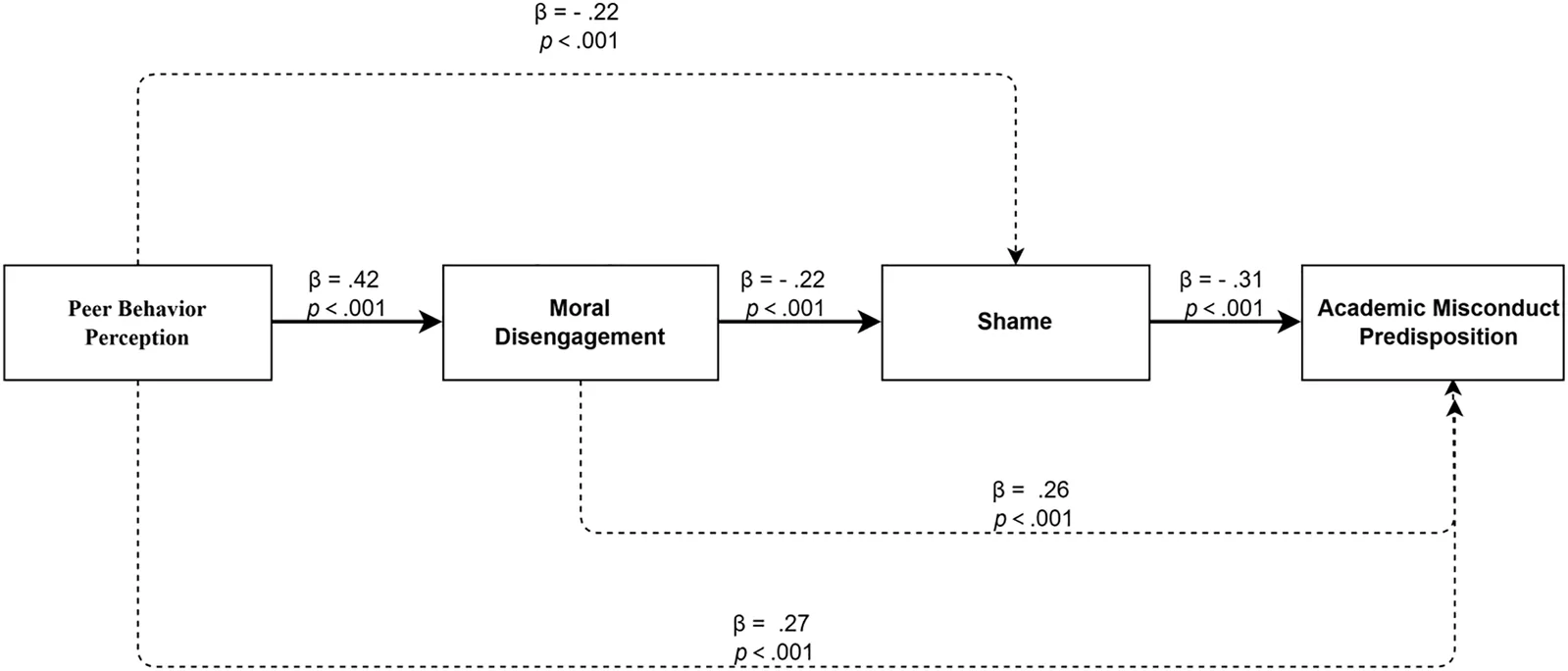
Serial mediation model linking peer behavior perception, moral disengagement, shame, and academic misconduct predisposition.

The Bootstrap indirect effect through moral disengagement was statistically significant, effect = .097, SE = .019, p < .001, 95% CI [.06, .14]. The indirect effect through shame was statistically significant, effect = .059, SE = .017, p < .001, 95% CI [.03, .09]. The serial indirect effect through moral disengagement and shame was statistically significant, effect = .026, SE = .008, p < .001, 95% CI [.01, .04]. The total indirect effect was .181, SE = .025, p < .001, 95% CI [.13, .23]. The direct effect was .238, SE = .039, p < .001, 95% CI [.16, .31], and the total effect was .418, SE = .037, p < .001, 95% CI [.35, .49].

### 3.5 Robustness check

The propensity score matching results are presented in Table 6. After matching, the standardized mean differences for the observed covariates were below .10. In the matched sample, students with higher peer behavior perception reported higher academic misconduct predisposition than students with lower peer behavior perception, effect = .401, SE = .077, *p* < .001.

**Table 6:** Propensity score matching (PSM).

Test Item / Variable	Pre-match Mean Difference (Treatment – Control)	Post-match Mean Difference (Treatment – Control)	Post-match Standardized Mean Difference (SMD)	Post-match T-test P-value
Covariate Balance				
Gender	− .04	− .02	− .04	> 0.1
Grade	− .017	− .04	− .03	> 0.1
Major	.013	.01	.02	> 0.1
GPA	−4.81	.04	.01	> 0.1
SES	− .05	− .01	− .01	> 0.1
Treatment Effect Estimation	Treatment Group Sample Size	Control Group Sample Size	Effect Estimate (SE)	Significance (P-value)
AM	158	158	.401 (.077)	< .001

Note. PSM = propensity score matching; SMD = standardized mean difference; ATT = average treatment effect on the treated. Values in parentheses are standard errors. The treatment group was defined as students with peer behavior perception scores above the median. Confidence intervals are reported with two decimal places where applicable.

The omitted-variable sensitivity analysis results are presented in Table 7. The robustness value for peer behavior perception was 27.18%. When academic performance was used as the benchmark covariate, the adjusted confidence interval under the three-times benchmark condition was [.09, .25].

**Table 7:** Sensitivity analysis for unobserved confounding variables.

Core Independent Variable	Original Effect Value (SE)	Partial R²	Robustness Value (RV_q = 1)	Significance Boundary (RV_q = 1, α = .05)	Extreme Confounder Bound (3x GPA strength)
PBP	.239 (.037)	9.21%	27.18%	19.81%	[.09, .25]

Note: The baseline model includes all control variables and mediators; the dependent variable is academic misconduct (AM), and the core independent variable is peer behavior perception (PBP). RV (Robustness Value) indicates the proportion of residual variance that an unobserved extreme confounder needs to explain to reduce the core effect to 0. The benchmark covariate is set to GPA. Values in parentheses are standard errors.

#### 3.5.1 Subsample robustness check.

The subsample results are presented in Table 8. The direct association between peer behavior perception and academic misconduct predisposition reached statistical significance across gender, grade-level, and disciplinary groups. The indirect effect through moral disengagement was also statistically significant across all subsamples. The indirect effect through shame reached statistical significance in most subsamples, except for the science group. The serial indirect effect through moral disengagement and shame was statistically significant in most subsamples, although the confidence interval in the science group did not clearly exclude zero after rounding. Overall, the subgroup results were generally consistent with the main model, while indicating some variation in indirect-effect estimates across disciplinary groups.

**Table 8:** Robustness test results for different subsamples.

Subsample	N	PBP → MD → AMP	PBP → SH → AMP	PBP → MD → SH → AMP	Direct effect	Total Direct
Gender grouping						
Male	216	0.11 [0.07, 0.17]	0.05 [0.02, 0.09]	0.02 [0.01, 0.04]	0.21 [0.08, 0.31]	0.18 [0.11, 0.23]
Female	202	0.08 [0.03, 0.13]	0.08 [0.02, 0.13]	0.03 [0.01, 0.06]	0.27 [0.17, 0.35]	0.19 [0.09, 0.29]
Grade grouping						
Lower grades (freshman and sophomore)	215	0.12 [0.07, 0.18]	0.06 [0.01, 0.12]	0.02 [0.01, 0.04]	0.15 [0.06, 0.26]	0.21 [0.14, 0.27]
Senior grades (junior and senior)	203	0.07 [0.04, 0.09]	0.06 [0.02, 0.11]	0.03 [0.01, 0.04]	0.35 [0.27, 0.45]	0.15 [0.10, 0.22]
Discipline grouping						
Liberal arts	126	0.13 [0.07, 0.20]	0.11 [0.01, 0.16]	0.03 [0.01, 0.08]	0.27 [0.15, 0.39]	0.27 [0.19, 0.33]
Science	148	0.12 [0.05, 0.17]	0.01 [-0.03, 0.04]	0.02 [-0.00, 0.03]	0.19 [0.10, 0.36]	0.15 [0.08, 0.20]
Engineering	144	0.05 [0.03, 0.13]	0.08 [0.02, 0.14]	0.03 [0.02, 0.05]	0.28 [0.12, 0.42]	0.17 [0.13, 0.25]

Note: Note. PBP = peer behavior perception; MD = moral disengagement; SH = shame; AMP = academic misconduct predisposition. Values are unstandardized effects, with 95% confidence intervals in brackets. Confidence intervals are reported with two decimal places. The exploratory interaction results are reported in S1 Appendix.

## 4 Discussion

### 4.1 Theoretical implications

The primary objective of this study was to examine how perceived peer misconduct is associated with students’ academic misconduct predisposition. The findings showed that peer behavior perception was positively associated with moral disengagement, moral disengagement was negatively associated with shame, and shame was negatively associated with academic misconduct predisposition. This pattern is consistent with Bandura’s moral disengagement theory, which regards unethical conduct as related not only to cognitive restructuring but also to the weakening of affective self-sanctions such as guilt and shame [16,22].

First, the positive association between peer behavior perception and academic misconduct predisposition is consistent with social cognitive theory and prior research on peer influence in academic integrity contexts [11,12,14]. When students perceive academic misconduct as common among peers, such perceptions may be associated with more permissive normative judgments regarding similar conduct. The present findings do not indicate observed misconduct behavior, but they suggest that perceived peer misconduct is related to students’ self-reported predisposition toward academic misconduct. This distinction is important because predispositions and attitudes are conceptually related to behavior, but they are not equivalent to verified behavioral outcomes.

Second, the findings clarify the role of moral disengagement in the academic misconduct context. Moral disengagement was positively associated with academic misconduct predisposition and also formed part of the indirect pathway from peer behavior perception to academic misconduct predisposition. This result is consistent with prior studies showing that moral disengagement is related to unethical conduct and academic cheating tendencies [18–20]. In academic settings, students who perceive misconduct as common among peers may be more likely to adopt rationalizations such as minimizing harm, diffusing responsibility, or viewing dishonest practices as normal responses to academic pressure. Such rationalizations may be associated with a greater self-reported predisposition toward academic misconduct.

Third, the findings suggest that shame can be examined within the moral disengagement process rather than only as a separate emotional pathway. This interpretation provides a more precise application of Bandura’s theory. Moral disengagement was negatively associated with shame, and shame was negatively associated with academic misconduct predisposition. These findings are consistent with the view that moral emotions can function as internal restraints in ethical judgment and conduct [24–26]. In this study, higher moral disengagement was associated with lower shame, and lower shame was associated with stronger academic misconduct predisposition. This pattern suggests that moral disengagement may be linked to academic misconduct predisposition partly through a reduced emotional cost attached to dishonest academic conduct.

Fourth, the significant serial indirect effect is consistent with a sequential association among peer behavior perception, moral disengagement, shame, and academic misconduct predisposition. The pathway from peer behavior perception to moral disengagement, from moral disengagement to lower shame, and from lower shame to academic misconduct predisposition was statistically significant. This finding is consistent with research suggesting that social norms, cognitive rationalization, and moral emotions are closely related in the domain of academic integrity [27–30]. Rather than presenting cognition and emotion as separate systems, the present study situates shame as an affective self-sanction within the moral disengagement framework.

The subgroup analyses also provide additional information about the stability and variation of the model across student groups. The direct association between peer behavior perception and academic misconduct predisposition reached statistical significance across gender, grade-level, and disciplinary groups. The indirect effect through moral disengagement was also found across the examined subsamples. Shame-related indirect effects and the serial indirect effect were generally consistent in most subsamples, although some disciplinary variation appeared, particularly in the science group, where some confidence intervals did not clearly exclude zero. These findings suggest that the general pattern of associations was similar across groups, while the relative contribution of specific indirect paths may vary across academic contexts.

### 4.2 Practical implications

The findings offer several implications for academic integrity education. Because peer behavior perception was associated with academic misconduct predisposition, universities may benefit from making academic integrity norms more visible and consistent. Integrity education should not focus only on punishment after misconduct occurs. It should also address students’ perceptions of peer norms, since students’ judgments about what peers commonly do may be related to their own academic misconduct predisposition. Clear communication of integrity expectations, transparent handling of misconduct cases, and peer-led academic integrity initiatives may help reduce the perception that dishonest practices are normal or widely tolerated.

The results also suggest the value of addressing moral disengagement in academic integrity programs. Students may justify dishonest academic practices by minimizing their consequences, shifting responsibility, or interpreting misconduct as a common response to academic pressure. Educational activities can therefore include case-based discussion, reflective writing, and guided analysis of common rationalizations for cheating or plagiarism. Such activities may help students identify how moral disengagement operates in academic contexts and how it is related to self-reported willingness to engage in misconduct.

In addition, the findings suggest that moral emotion should be handled carefully in academic integrity education. Shame was negatively associated with academic misconduct predisposition, but this does not imply that institutions should use public shaming or stigmatizing practices. A more appropriate approach is to cultivate reflective awareness of the personal, relational, and institutional consequences of academic dishonesty. Case-based learning can help students consider how dishonest academic conduct affects classmates, teachers, academic evaluation, and professional development. This approach may support moral reflection without relying on punitive or humiliating forms of emotional pressure.

The subgroup results also suggest that integrity education may need to be adapted to different student groups. Since the size and statistical significance of some indirect effects varied across grade levels and disciplines, academic integrity programs may be more effective when they are connected to students’ disciplinary learning environments and academic tasks. For example, writing-intensive courses may emphasize plagiarism and citation ethics, while laboratory or engineering courses may emphasize data integrity, collaboration boundaries, and responsibility in technical work. Such discipline-sensitive approaches may make academic integrity education more relevant to students’ actual learning contexts.

### 4.3 Limitations and future research directions

First, the study used a cross-sectional survey design. The hypothesized direction of the model was based on moral disengagement theory and prior research, but the data do not establish temporal order. The associations among peer behavior perception, moral disengagement, shame, and academic misconduct predisposition should therefore be interpreted as cross-sectional associations. Future research could use longitudinal or experimental designs to examine whether changes in perceived peer misconduct are followed by changes in moral disengagement, shame, and academic misconduct predisposition over time.

Second, the outcome variable measured academic misconduct predisposition rather than verified academic misconduct behavior. Participants reported their tendency or likelihood to engage in academic misconduct, not whether they were observed or officially recorded as committing misconduct. This distinction is important because predisposition, intention, and attitude are related to but not identical to actual behavior. Future studies could combine self-report measures with behavioral indicators, institutional records, scenario-based behavioral tasks, or longitudinal follow-up data to examine how academic misconduct predisposition is related to later conduct.

Third, the study relied on self-report data. Although anonymous data collection and common method bias diagnostics were used, self-report measures of academic dishonesty and moral emotions may still be influenced by social desirability and individual differences in self-presentation. Future research could include multi-source data, peer reports, teacher reports, or behavioral measures to provide a broader assessment of academic integrity-related processes.

Fourth, the data were collected from one comprehensive university in Shandong Province. The sample included students from different grade levels and disciplinary categories, and the demographic composition was reported descriptively. Since university-wide enrollment benchmarks were not available for direct comparison, the representativeness of the sample should be interpreted within the study context. Future studies could use multi-university samples and institutional population data to examine whether the model shows similar patterns across regions, institutional types, and academic disciplines.

Fifth, the robustness checks provide supplementary information but do not change the observational nature of the study. Propensity score matching addressed observed covariate balance, and omitted-variable sensitivity analysis provided information about the degree of unobserved confounding needed to alter the main association. Future research could extend the current model by including additional individual and contextual variables, such as moral identity, institutional integrity climate, academic pressure, and perceived enforcement of academic integrity policies.

## 5 Conclusion

This study examined the association between peer behavior perception and academic misconduct predisposition through a serial mediation model involving moral disengagement and shame. The findings suggest that students who perceived higher levels of peer misconduct reported higher moral disengagement, which was associated with lower shame and higher academic misconduct predisposition. The serial indirect effect through moral disengagement and shame was statistically significant, indicating that shame may function as an affective self-sanction within the moral disengagement process.

The study contributes to the academic integrity literature by providing a theory-consistent account of moral disengagement in relation to academic misconduct predisposition. Rather than treating moral disengagement as only a cognitive mechanism and shame as a separate emotional mechanism, the present study places shame within the broader process of moral self-regulation. The findings also suggest that academic integrity education may benefit from addressing perceived peer norms, common rationalizations for dishonest conduct, and reflective awareness of moral emotions. Future research using longitudinal, experimental, multi-source, and multi-institutional designs can further examine the temporal and behavioral implications of these associations.

## Supporting information

S1 Appendix Hierarchical regression analysis of the interaction effect of moral disengagement and shame. (DOCX)

## References

[1] Li X, Zhao J, Yan W. Integrity levels of Chinese college students: An analysis of influencing factors. Front Educ. 2022;7. doi: 10.3389/feduc.2022.843674

[2] Wang H, Zhang Y. The effects of personality traits and attitudes towards the rule on academic dishonesty among university students. Sci Rep. 2022;12(1):14181. doi: 10.1038/s41598-022-18394-3 35986157 PMC9390108

[3] Yang SC, Chiang FK, Huang CL. A comparative study of academic dishonesty among university students in Mainland China and Taiwan. Asia Pacific Educ Rev. 2017;18(3):385–99. doi: 10.1007/s12564-017-9497-2

[4] Chapman KJ, Davis R, Toy D, Wright L. Academic Integrity in the Business School Environment: I’ll Get by with a Little Help from My Friends. Journal of Marketing Education. 2004;26(3):236–49. doi: 10.1177/0273475304268779

[5] Veríssimo AC, Conrado GAM, Barbosa J, Gomes SF, Severo M, Oliveira P, et al. Machiavellian Medical Students Report More Academic Misconduct: A Cocktail Fuelled by Psychological and Contextual Factors. Psychol Res Behav Manag. 2022;15:2097–105. doi: 10.2147/PRBM.S370402 35983023 PMC9380600

[6] Brimble M, Stevenson-Clarke P. Perceptions of the prevalence and seriousness of academic dishonesty in Australian universities. Aust Educ Res. 2005;32(3):19–44. doi: 10.1007/bf03216825

[7] Bin-Nashwan SA, Sadallah M, Bouteraa M. Use of ChatGPT in academia: Academic integrity hangs in the balance. Technology in Society. 2023;75:102370. doi: 10.1016/j.techsoc.2023.102370

[8] Kelley PC, Chang PL. A Typology of University Ethical Lapses: Types, Levels of Seriousness, and Originating Location. The Journal of Higher Education. 2007;78(4):402–29. doi: 10.1080/00221546.2007.11772322

[9] Krou MR, Fong CJ, Hoff MA. Achievement Motivation and Academic Dishonesty: A Meta-Analytic Investigation. Educ Psychol Rev. 2020;33(2):427–58. doi: 10.1007/s10648-020-09557-7

[10] Wu C. Publish or perish: a study on academic misconduct in publishing among Chinese doctoral students. British Journal of Sociology of Education. 2025;46(3):303–22. doi: 10.1080/01425692.2025.2454315

[11] Bandura A. Social cognitive theory of moral thought and action. In: Kurtines WM, Gewirtz JL, editors. Handbook of moral behavior and development. Lawrence Erlbaum Associates. 1991. p. 45–103. doi: 10.4324/9780203052457

[12] McCabe DL, Trevino LK, Butterfield KD. Cheating in Academic Institutions: A Decade of Research. Ethics & Behavior. 2001;11(3):219–32. doi: 10.1207/s15327019eb1103_2

[13] Aguilar-Pardo D, Martínez-Fernández B, Colmenares F, Martín-Babarro J. Peer likeability and victimization in young adolescents: Moderating effects of descriptive and status group norms for aggression and prosocial behaviour and network density. Br J Educ Psychol. 2022;92(3):925–54. doi: 10.1111/bjep.12481 35032033

[14] Zhao L, Mao H, Compton BJ, Peng J, Fu G, Fang F, et al. Academic dishonesty and its relations to peer cheating and culture: A meta-analysis of the perceived peer cheating effect. Educational Research Review. 2022;36:100455. doi: 10.1016/j.edurev.2022.100455

[15] Bandura A. Selective Moral Disengagement in the Exercise of Moral Agency. Journal of Moral Education. 2002;31(2):101–19. doi: 10.1080/0305724022014322

[16] Bandura A. Moral disengagement: How people do harm and live with themselves. New York: Worth Publishers. 2016.

[17] Zhang L, Amos C, Pentina I. Interplay of rationality and morality in using ChatGPT for academic misconduct. Behaviour & Information Technology. 2024;44(3):491–507. doi: 10.1080/0144929x.2024.2325023

[18] Stephens JM, Nicholson H. Cases of incongruity: exploring the divide between adolescents’ beliefs and behavior related to academic dishonesty. Educational Studies. 2008;34(4):361–76. doi: 10.1080/03055690802257127

[19] Moore C, Detert JR, Klebe Treviño L, Baker VL, Mayer DM. Why employees do bad things: moral disengagement and unethical organizational behavior. Personnel Psychology. 2012;65(1):1–48. doi: 10.1111/j.1744-6570.2011.01237.x

[20] Fida R, Tramontano C, Paciello M, Ghezzi V, Barbaranelli C. Understanding the Interplay Among Regulatory Self-Efficacy, Moral Disengagement, and Academic Cheating Behaviour During Vocational Education: A Three-Wave Study. J Bus Ethics. 2016;153(3):725–40. doi: 10.1007/s10551-016-3373-6

[21] Huang CL, Yang SC, Yang CA. Exploration of students’ perception of academic misconduct: Do individual factors, moral philosophy, behavioral intention, and judgment matter?. Frontiers in Psychology. 2022;13:857943. doi: 10.3389/fpsyg.2022.857943 PMC9017647 35450332

[22] Bandura A, Barbaranelli C, Caprara GV, Pastorelli C. Mechanisms of moral disengagement in the exercise of moral agency. Journal of Personality and Social Psychology. 1996;71(2):364–74. doi: 10.1037/0022-3514.71.2.364

[23] Lewis HB. Shame and guilt in neurosis. New York: International Universities Press. 1971.

[24] Tangney JP, Dearing RL. Shame and guilt. Guilford Press. doi: 10.4135/9781412950664.n388

[25] Curtis GJ. Guilt, Shame and Academic Misconduct. J Acad Ethics. 2023;1–15. doi: 10.1007/s10805-023-09480-w 37362772 PMC10164613

[26] Haidt J. The Moral Emotions. Handbook of Affective Sciences. Oxford University PressNew York, NY. 2002. p. 852–70. doi: 10.1093/oso/9780195126013.003.0045

[27] Gino F, Ayal S, Ariely D. Contagion and differentiation in unethical behavior: the effect of one bad apple on the barrel. Psychol Sci. 2009;20(3):393–8. doi: 10.1111/j.1467-9280.2009.02306.x 19254236

[28] Culiberg B, Mihelič KK. The impact of mindfulness and perceived importance of peer reporting on students’ response to peers’ academic dishonesty. Ethics & Behavior. 2019;30(5):385–99. doi: 10.1080/10508422.2019.1628644

[29] Stephens JM. How to Cheat and Not Feel Guilty: Cognitive Dissonance and its Amelioration in the Domain of Academic Dishonesty. Theory Into Practice. 2017;56(2):111–20. doi: 10.1080/00405841.2017.1283571

[30] Yu L. The Positive Motivation of Shame: Moral Emotion in the Mencius. Religions. 2023;14(4):495. doi: 10.3390/rel14040495

[31] Brislin RW. Translation and content analysis of oral and written material. In: Triandis HC, Berry JW, editors. Handbook of cross-cultural psychology: Methodology. Boston: Allyn and Bacon. 1980. p. 389–444.

[32] McCabe DL, Trevino LK. Individual and Contextual Influences on Academic Dishonesty: A Multicampus Investigation. Research in Higher Education. 1997;38(3):379–96. doi: 10.1023/a:1024954224675

[33] Yang SC, Huang C-L, Chen A-S. An Investigation of College Students’ Perceptions of Academic Dishonesty, Reasons for Dishonesty, Achievement Goals, and Willingness to Report Dishonest Behavior. Ethics & Behavior. 2013;23(6):501–22. doi: 10.1080/10508422.2013.802651

[34] McCabe DL. Cheating among college and university students: A North American perspective. IJEI. 2005;1(1). doi: 10.21913/ijei.v1i1.14

[35] Rettinger DA, Kramer Y. Situational and Personal Causes of Student Cheating. Res High Educ. 2008;50(3):293–313. doi: 10.1007/s11162-008-9116-5

[36] Cohen TR, Wolf ST, Panter AT, Insko CA. Introducing the GASP scale: a new measure of guilt and shame proneness. J Pers Soc Psychol. 2011;100(5):947–66. doi: 10.1037/a0022641 21517196

