# Supplementary material for: How peer behavior shapes academic misconduct: The roles of moral disengagement and shame
Source: PLoS One. 2026 Oct 14;21(10):e0358871. doi: 10.1371/journal.pone.0358871 (PMC13667821; doi:10.1371/journal.pone.0358871)
Supplement: Supplementary file 1 [file pone.0358871.s001.docx]

Appendix Table A1

Table A1 Hierarchical Regression Analysis of the Interaction Effect of Moral Disengagement and Shame

| Variable | (1) | (2) | (3) |
| --- | --- | --- | --- |
| Control variable |  |  |  |
| Gender | 0.001  （0.005） | -0.010  （-0.163） | 0.002  （0.027） |
| Grade | 0.107**  （3.053） | 0.043  （1.533） | 0.046  （1.643） |
| Major type | -0.021  （-0.381） | -0.025  （-0.581） | -0.024  （-0.576） |
| Academic performance | -0.017***  （-4.498） | -0.006*  （-2.176） | -0.006*  （-2.184） |
| Main effect |  |  |  |
| Peer Behavior Perception |  | 0.238***（6.397） | 0.238***（6.473） |
| Moral Disengagement |  | 0.255***（6.181） | 0.235***（5.702） |
| Shame |  | -0.285***  （-7.764） | -0.286***  （-7.883） |
| Interaction effect |  |  |  |
| Moral Disengagement × Shame | |  | -0.114**  （-3.068） |
| Model statistics | |  |  |
| R² | 0.082 | 0.459 | 0.471 |
| ΔR² | 0.082 | 0.376 | 0.012 |
| F | 9.256 | 49.639 | 45.502 |

Note: *p < 0.05, **p < 0.01, ***p < 0.001. Values in parentheses are t values. The interaction analysis was conducted as an exploratory supplementary analysis and was not included in the main serial mediation model.
